# Flexible and High Performance Supercapacitors Based on NiCo_2_O_4_for Wide Temperature Range Applications

**DOI:** 10.1038/srep15265

**Published:** 2015-10-20

**Authors:** Ram K. Gupta, John Candler, Soubantika Palchoudhury, Karthik Ramasamy, Bipin Kumar Gupta

**Affiliations:** 1 Department of Chemistry, Pittsburg State University, 1701 S. Broadway, Pittsburg, KS 66762, USA; 2 Center for Materials for Information Technology, The University of Alabama, Tuscaloosa, AL 35487, USA; 3 Center for Integrated Nanotechnologies, Los Alamos National Laboratory, Albuquerque, NM 87545, USA; 4 CSIR -National Physical Laboratory, Dr K.S. Krishnan Road, New Delhi 110012, India

## Abstract

Binder free nanostructured NiCo_2_O_4_ were grown using a facile hydrothermal technique. X-ray diffraction patterns confirmed the phase purity of NiCo_2_O_4_. The surface morphology and microstructure of the NiCo_2_O_4_ analyzed by scanning electron microscopy (SEM) showed flower-like morphology composed of needle-like structures. The potential application of binder free NiCo_2_O_4_ as an electrode for supercapacitor devices was investigated using electrochemical methods. The cyclic voltammograms of NiCo_2_O_4_ electrode using alkaline aqueous electrolytes showed the presence of redox peaks suggesting pseudocapacitance behavior. Quasi-solid state supercapacitor device fabricated by sandwiching two NiCo_2_O_4_ electrodes and separating them by ion transporting layer. The performance of the device was tested using cyclic voltammetry, galvanostatic charge-discharge and electrochemical impedance spectroscopy. The device showed excellent flexibility and cyclic stability. The temperature dependent charge storage capacity was measured for their variable temperature applications. Specific capacitance of the device was enhanced by ~150% on raising the temperature from 20 to 60 °C. Hence, the results suggest that NiCo_2_O_4_ grown under these conditions could be a suitable material for high performance supercapacitor devices that can be operated at variable temperatures.

With increasing demand for energy and limited fossil fuels, there is an unprecedented urgency in developing high performance and stable materials for energy related applications^1^,^2^,^3^,^4^,^5^,^6^,^7^,^8^. Albeit a significant progress has been made in recent years for the development of cost effective and better performing materials for energy conversion and storage applications, it still lags behind in meeting the global demand. Supercapacitors are the emerging energy storage technology, attractive primarily because of their ability to store and release energy instantaneously, long life, lower cost and high power density^9^,^10^,^11^,^12^. It has found use in electric vehicles, consumer electronics, portable devices and wherever a burst of energy supply is required^13^,^14^,^15^,^16^,^17^. The material systems that have majorly been encompassed for supercapacitor applications are carbon based materials, conducting polymers and metal oxides^1^,^14^,^16^,^18^,^19^,^20^. Among them, metal oxides are appealing as they exhibit large energy and power densities. Despite many binary metal oxides (*e.g.,* RuO_2_, NiO, Co_3_O_4_, Mo_2_O_3_, V_2_O_5_ and MnO_2_) displaying promising performances, ternary or higher order metal oxides are particularly attractive for supercapacitor applications^17^,^21^,^22^,^23^,^24^,^25^,^26^,^27^,^28^. Ternary or higher order metal oxides provide additional redox sites for pseudocapacitance reactions and thereby offer an opportunity for enhancing the specific capacitance values^29^.

NiCo_2_O_4_ is a ternary oxide material containing mixed valence metals and allows multiple electrochemical processes^30^. In addition, growing nanocrystalline form of NiCo_2_O_4_ directly on the conducting surface, the performance of supercapacitor devices can be augmented^31^. By effecting this growth on conductive substrates, each nanostructured metal oxide gets its own electric contact with the substrate and thus, participates in the electrochemical reactions more efficiently. Accordingly, it has been investigated for supercapacitor applications to some extent in recent years. Huang *et al.* have fabricated electrodes using a facile electrodeposition of cobalt and nickel double hydroxide nanosheets on porous NiCo_2_O_4_ nanowires grown on carbon fiber paper^32^. These materials showed very good cycling stability with a high capacitance of ∼1.64 F/cm^2^ at 2 mA/cm^2^. Furthermore, these electrodes exhibited excellent rate capability and retained 74% of the capacitance by increasing current density from 2 to 90 mA/cm^2^.

Metallic substrates such as nickel foam and titanium sheets have also used for the preparation of NiCo_2_O_4_ for supercapacitor applications^33^. The areal capacitances as high as 3.12, 1.44, 0.99,0.79 and 0.59 F/cm^2^ were observed at discharge current densities of 1.11, 2.78, 5.56, 11.12 and 22.24 mA/cm^2^, respectively for NiCo_2_O_4_ on nickel electrode. Qian *et al.* have used a facile electrodeposition technique to synthesize NiCo_2_O_4_ on a stainless steel and indium tin oxide electrode^34^. These electrodes were used in the electrocatalytic oxidation of methanol. The NiCo_2_O_4_ showed much higher electrocatalytic activity, lower over potential and greater stability compared to that of only NiO or Co_3_O_4_ synthesized by the same method. Hierarchical NiCo_2_O_4_@NiCo_2_O_4_ core/shell nanoflake arrays were grown on nickel foam using a two-step solution-based method^35^. It was reported that the core/shell electrode showed better pseudocapacitive behaviors compared with the bare NiCo_2_O_4_ nanoflake. The maximum areal specific capacitance of 2.20 F/cm^2^ at a current density of 5 mA/cm^2^ was observed which was retained at about 98.6% of the initial value after 4000 cycles.

Recently, we have grown highly porous hierarchical flexible nanosheets of NiCo_2_O_4_-graphene oxide (NiCo_2_O_4_-GO) on nickel foam using a facile electrochemical deposition method^31^. The electrochemical testing revealed a very high a specific capacitance of 1078 F/g at a discharge current of 1 mA with very good cyclic stability. These excellent capacitive performances were attributed to the hierarchical porous nanosheet like structures of NiCo_2_O_4_-GO. Despite these investigations, the performance of NiCo_2_O_4_ supercapacitors at variable temperatures has not been studied. Advancing our interest on this material for supercapacitor applications, herein we report a facile hydrothermal method to grow flower-like morphology consisting needle-like structures. The electrochemical performance of NiCo_2_O_4_ electrode showed a specific capacitance of 845 F/g in 3 M KOH electrolyte with excellent cyclic stability. The specific capacitance of supercapacitor device fabricated using these electrodes is estimated to be 77 F/g in 3 M KOH at room temperature. Flexibility studies of the device carried out by bending the device at different angles showed no reduction in specific capacitance values. Furthermore, the specific capacitance of NiCo_2_O_4_ based supercapacitor device at different temperatures is estimated to be increasing linearly on raising the temperature.

## Experimental Details

Nanostructured NiCo_2_O_4_ was synthesized over nickel foam using a hydrothermal method. For this, analytical grade Ni(NO_3_)_2_.6H_2_O and Co(NO_3_)_2_.6H_2_O were used. Before synthesis, the nickel foam was cleaned in 3 M HCl using a bath sonicator followed by cleaning in deionized water. The nickel foam was again cleaned in isopropanol and acetone, respectively. The nickel foam was completely dried before measuring its weight. In a typical synthesis of NiCo_2_O_4_, 1 mmol of Ni(NO_3_)_2_.6H_2_O and 2 mmol of Co(NO_3_)_2_.6H_2_O were dissolved in 8 ml of water/ethanol (1:1 v/v) solution using sonication. In another beaker, 300 mg of polyvinylpyrrolidone (PVP) was dissolved in 10 ml DI water/ethanol (1:1 v/v). PVP solution was added slowly to the above solution under sonication. Into this mixture, 12 mmol (dissolved in 18 ml water/ethanol (1:1 v/v)) of urea was added under stirring and sonication. The entire solution was transferred to a 45 mL Teflon lined autoclave having the pre cleaned and weighed nickel foam. The autoclave was maintained at 140 °C for 12 hrs and then cooled to room temperature naturally. The nickel foam was taken out and washed several times with distilled water and absolute ethanol. The nickel foam was dried at 60 °C for 8 hrs and finally at 350 °C for 3 hrs.

The structural characterization and surface morphology of NiCo_2_O_4_ was studied using X-ray diffraction (XRD), scanning electron microscopy (SEM) and transmission electron microscopy (TEM). The XRD spectra was recorded with Shimadzu X-ray diffractometer using the 2*θ-θ* scan with CuK_α1_ (λ = 1.5406 Å) radiation. Scanning electron microscope (SEM) imaging and EDX mapping analysis were carried out using a JEOL 7000 FE SEM equipped with energy dispersive X-ray spectroscopy (EDX), wavelength dispersive X-ray spectroscopy (WDS), electron backscatter diffraction (EBSD), secondary electron (SE), backscattered electron (BE) and transmission electron (TE) detectors. Transmission electron microscopy (TEM) analysis was performed using a FEI-Tecnai, 200 kV transmission electron microscope equipped with a CCD camera for STEM, HAADF detector, and EDX. TEM image non-linear processing was carried out using Gatan digital micrograph version 3.4.

Standard three electrode cells were used for electrochemical testing of the samples. A platinum wire (as a counter electrode), saturated calomel electrode (as a reference electrode) and NiCo_2_O_4_ deposited nickel foam (as a working electrode) were used for electrochemical measurements. An aqueous solution containing 3 M KOH, NaOH and LiOH were used as electrolytes. The flexible quasi-solid state (using liquid electrolyte) device was assembled using two working electrodes separated by ion transporting layer (Celgard, 25μm thick, 39% porosity) in KOH electrolyte. Before assembling the supercapacitor device, both working electrodes and ion transporting layer were soaked in the KOH electrolyte for 1 hr. The charge storage capacity of the electrode and device was studied using cyclic voltammetry (CV) and galvanostatic charge-discharge methods. Electrochemical impedance spectroscopy (EIS) measurements were carried out by applying an AC voltage with 10 mV amplitude in a frequency range from 0.05 Hz to 10 kHz at open circuit potential. Electrochemical measurements were performed on a VersaSTAT 4–500 electrochemical workstation (Princeton Applied Research, USA).

## Results and Discussion

**Figure 1:**
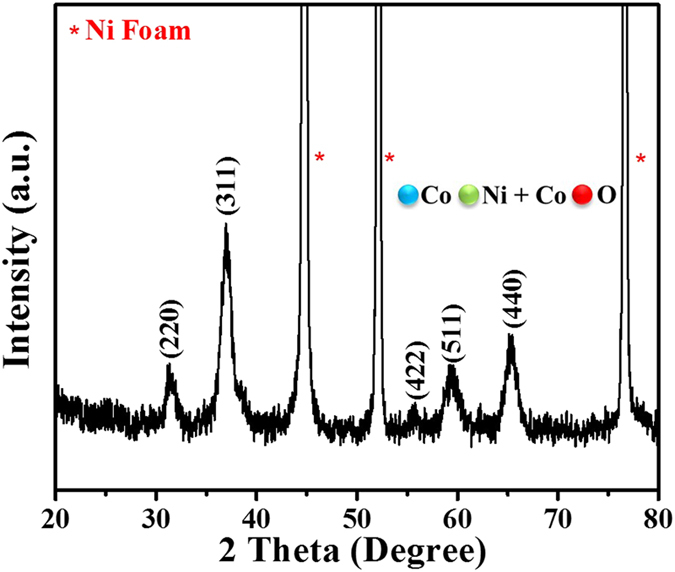
XRD pattern of NiCo_2_O_4_ nanostructures grown on Ni foam.

Binder free electrodes decorated with nanocrystals of NiCo_2_O_4_ on nickel foam were prepared following a facile hydrothermal method. We have chosen the hydrothermal method for the growth of NiCo_2_O_4_ on nickel foam, because it is known for growth of phase pure materials with hierarchical structures. Creation of a pressurized condition in hydrothermal synthesis is beneficial for the metal salts to react with polymer molecules and transform to the desired metal oxide nanostructures. Moreover, the nickel foam in the hydrothermal vessel acts as a substrate for the metal salts to reside and react with the polymer and thereby leads to the growth of nanostructures at the surface. We have analyzed crystallinity and phase purity of the synthesized NiCo_2_O_4_ nanostructures using powder X-ray diffraction. X-ray diffraction pattern of the hydrothermally synthesized NiCo_2_O_4_ over nickel foam is shown in Fig. 1. The observed X-ray diffractions peaks are found to be intense and broad indicating that the nature of NiCo_2_O_4_ is highly crystalline and comprises nano-sized crystallites. The diffraction peak positions match well with standard diffraction pattern and the reflections can be indexed to cubic phase of NiCo_2_O_4_ (JCPDS 20–0781). We did not observe any additional peaks arising from the binary phases of oxides of nickel or cobalt, indicating the sample is free of any crystalline impurities. The major diffraction peaks are indexed as (111), (311), (440), (222) planes of spinel phase NiCo_2_O_4_.

**Figure 2:**
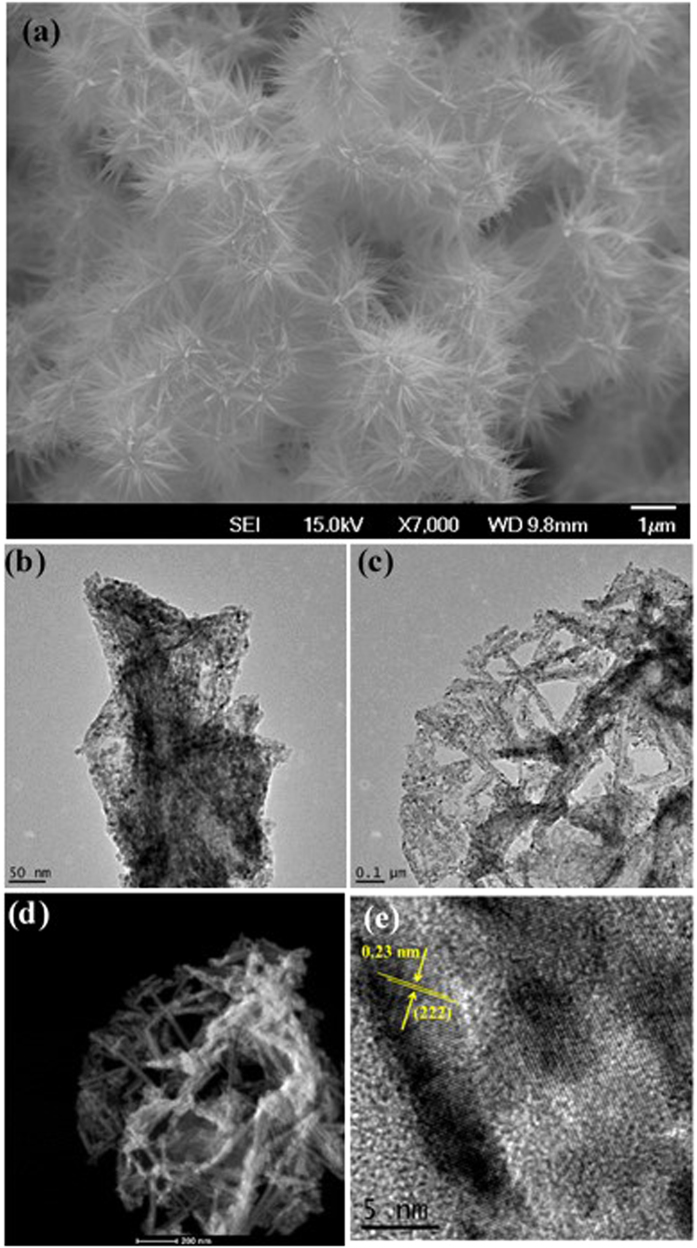
(**a**) SEM image, (**b**,**c**) TEM images, (**d**) HAADF image, and (**e**) HRTEM image of NiCo_2_O_4_ nanocrystals grown on Ni foam.

Morphology of the synthesized NiCo_2_O_4_ on nickel foam has been analyzed using scanning electron microscopy (SEM). The SEM image of the NiCo_2_O_4_ grown on nickel foam is shown in Fig. 2(a). The image shows flower like morphology composed of needles, which was observed over the entire nickel foam confirming the uniform growth of the crystallites. The diameter of the needle-like structures is estimated to be ranging from 15 to 20 nm with lengths of several hundred nanometers. Elemental composition of NiCo_2_O_4_ grown on nickel foam was confirmed using energy dispersive X-ray spectrometer (EDX). EDX analysis showed the presence of Ni, Co and O with an average ratio of 1:2 for Ni: Co (Fig. 1S). Further to gather insight of the NiCo_2_O_4_ nanostructure, we have carried out transmission electron microscopy (TEM) imaging from the sample obtained by sonicating NiCo_2_O_4_ grown on nickel foam. TEM images in Fig. 2(b,c) show leaf-like branched structures that are composed of smaller nanocrystals. In addition, we have carried out high angle annular dark field imaging (HAADF) that provide information about atomic number (*Z*) contrast. The HAADF image in Fig. 2(d) of our sample shows uniform contrast suggesting the homogenous distribution of elements. High resolution transmission electron microscope (HRTEM) image in Fig. 2(e) shows lattice fringes from the crystallites. The measured lattice spacing between the fringes is 0.23 nm corresponding to (222) plane of cubic phase NiCo_2_O_4_.

**Figure 3:**
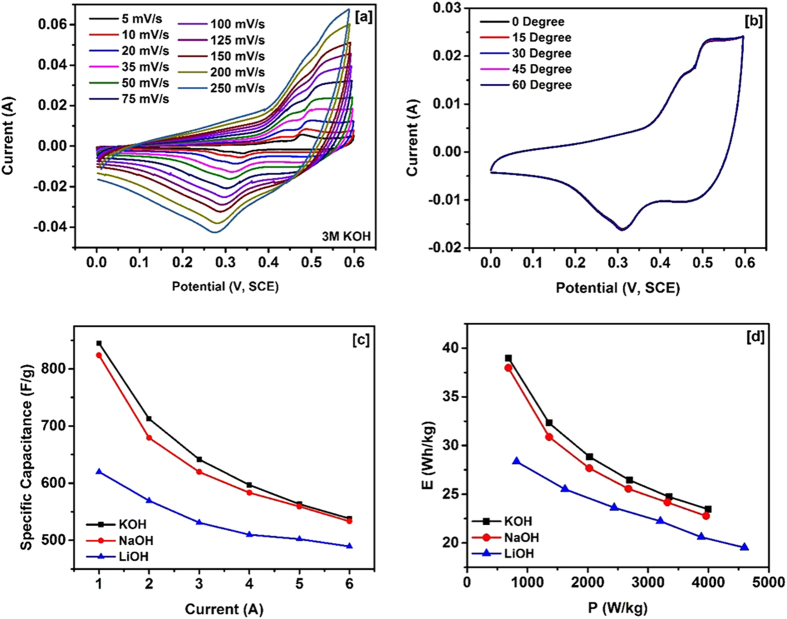
(**a**) CV curves of NiCo_2_O_4_ at various scan rates in KOH electrolyte, (**b**) CV curves of NiCo_2_O_4_ at various bending angles in KOH, (**c**) Charge-discharge characteristics of NiCo_2_O_4_ in various electrolytes, and (**d**) Ragone plots of NiCo_2_O_4_ in various electrolytes.

In order to evaluate the binder free NiCo_2_O_4_ nanostructures for supercapacitor applications, we have carried out systematic electrochemical investigations on the electrode and the quasi-solid state device fabricated using NiCo_2_O_4_ electrodes. Cyclic voltammograms of the NiCo_2_O_4_ electrode using three different electrolytes such as LiOH, NaOH and KOH are given in Figure 2S. We have determined that the highest current density and area under the CV curve was obtained from the KOH electrolyte. As seen in the Figure 2S, the redox peaks using three different electrolytes occur nearly at the same potentials, while the redox currents are different. This could be due to ionic size and thus mobility of the electrolytes used. In the aqueous solution, hydrated radius of Li^+^ ions is the highest and expected to have the lowest mobility and thus the redox current in comparison with Na^+^ and K^+^ ions. Figure 3(a) shows the CV curves of NiCo_2_O_4_ in 3 M KOH electrolyte at various scan rates. Pairs of redox wave are apparent from the CV curves indicating pseudocapacitance behavior of the material with redox waves attributing for Co (II)/Co (III) and Ni(II)/Ni(III) redox couples^35^. The shape and redox potentials of the CV curves are comparable to those reported for NiCo_2_O_4_^36^,^37^. From the CV curves at various scan rates, it can be noticed that the peak current increases with increasing the scan rate and difference in the cathodic and anodic peak potential expands gradually, indicating diffusion controlled reaction kinetics^38^. The potential application of the hydrothermally grown NiCo_2_O_4_nanostructures on nickel foam as flexible binder free electrode was examined by measuring CV curves at various bending angles (Fig. 3b). As seen in the flexibility test, the CV curves of the electrode at various bending angles are identical in shape, indicating high electrochemical bending stability.

The charge storage capacity of the NiCo_2_O_4_ electrode was further investigated using galvanostatic charge-discharge measurements. The charge-discharge curves were observed to be symmetrical in nature (Fig. 3S), suggesting high electrochemical reversibility and fast reaction kinetics of NiCo_2_O_4_ electrode^13^,^39^. In addition to the symmetrical nature, a potential platform was observed in charge-discharge curves, indicating typical pseudocapacitance behavior of the electrode. This could be due to charge transfer reaction or electrochemical adsorption/desorption process at the electrode/electrolyte interface. The specific capacitance (*C*_*sp*_) of the NiCo_2_O_4_ electrode was calculated using the equation^40^:
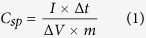
where, *I* is the discharge current (*A*), Δ*t* is the discharge time (s), Δ*V* is the potential window (V), and *m* is the mass (g) of the NiCo_2_O_4_. Figure 3c shows the variation of specific capacitance versus discharge current for the NiCo_2_O_4_ electrode in various electrolytes. As seen, the specific capacitance of the electrode decreases with increasing current for all electrolytes. The decrease in the specific capacitance with the increasing discharge current could be due to increase in potential drop and insufficient faradic redox reaction at higher currents. The energy density and the power density for the NiCo_2_O_4_ electrode was calculated using the expressions^41^:
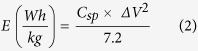

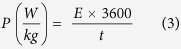
where *C*_*sp*_ (F/g) is the specific capacitance calculated from charge-discharge characteristics, Δ*V* (V) is the potential window and *t* (s) is the discharge time. The variations of the specific energy density versus power density (Ragone plot) for different electrolytes are shown in Fig. 3d. The highest specific capacitance (845 F/g), specific energy density (39 Wh/kg) and power density (4000 W/kg) was observed for the KOH electrolyte. The estimated energy and power density values are comparable to the values reported for NiCo_2_O_4_ in the literature^42^,^43^. Table 1 compares the specific capacitance of NiCo_2_O_4_ nanostructures obtained in this work with the values reported for various morphologies of NiCo_2_O_4_ synthesized by the hydrothermal method. In Table 2, we have compared the properties of NiCo_2_O_4_ grown on different substrates using various methods. As seen in the table, the higher specific capacitance are generally observed for the substrates having nanostructures such as nickel foam and carbon fibers. The higher specific capacitance on such substrates are due to nanostructure NiCo_2_O_4_ having direct contact with the conducting substrates. At this juncture, it is worthy to compare the electrochemical properties NiCo_2_O_4_ with one of the highly studied, but expensive metal oxide supercapacitor materials RuO_2_ and its carbon composites. Chen *et al.* reported the hydrothermal synthesis of RuO_2_ and reduced graphene oxide (RGO) composites^44^. Electrochemical measurements of the composites with 45 wt% of RuO_2_showed specific capacitance value of 471 F/g at 0.5 A/g with 92% capacitance retention after 3000 cycles. A similar RuO_2_/RGO composites synthesized using poly(diallyl dimethyl ammonium chloride) (PDDA) exhibited a specific capacitance value of ~540 F/g for 27.5% Ru loading at 5 mV/s^45^. Recently, Lin *et al.* synthesized RuO_2_/graphene sheets with different Ru contents by hydrothermal method^46^. The estimated specific capacitance value from the electrochemical measurements of these RuO_2_/graphene composites (40 wt% Ru) is found to be 551 F/g at 1 A/g in 1 M H_2_SO_4_. Recently, Wang *et al.* have used a three-dimensional (3D) sub-5 nm hydrous RuO_2_ anchored graphene and CNT hybrid foam (RGM) architecture for high-performance supercapacitor electrodes^47^. The supercapacitor based on this unique structured hybrid material showed a high specific capacitance of 502.78 F/g (areal capacitance of 1.11 F/cm^2^) with an energy density of 39.28 Wh/kg and power density of 128.01 kW/kg. The molecular level interaction of single crystalline RuO_2_ with electrolyte ions has been determined in detail using surface X-ray scattering measurements coupling with electrochemical measurements^48^,^49^. Authors have concluded that the ordering of water on the surface of RuO_2_ is responsible for electrocatalytic behavior of RuO_2_. It was observed that the two different faces, (110) and (100), showed different cyclic voltammograms. Based on this, we postulate that the similar molecular level interaction may also occur when NiCo_2_O_4_ is exposed to aqueous electrolyte solution.

**Figure 4:**
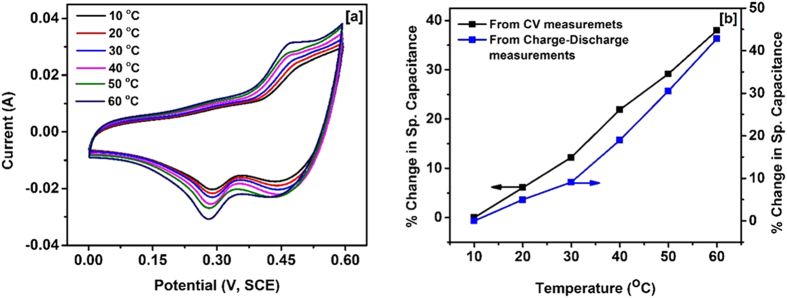
(**a**) CV curves of NiCo_2_O_4_ at different temperatures, and (**b**) Plots showing changes in specific capacitance values at different temperatures.

It is important to study the electrochemical properties of the materials at different temperatures in order to develop supercapacitor devices that can be used in a wide temperature range. For that reason, we have carried out cyclic voltammetry measurements at the temperature range of 10–60 °C. CV curves of the electrode measured at various temperatures are shown in Fig. 4a. We have observed increase in the area under the CV curves and the discharge time in galvanostatic charge-discharge measurements by raising the temperature from 10 to 60 °C, indicating the improvement in the charge storage capacity of NiCo_2_O_4_ electrode (Fig. 4S). From the CV and galvanostatic charge-discharge measurements, it was observed that charge storage capacity of NiCo_2_O_4_ electrode betters by about 40% by increasing temperature from 10 °C to 60 °C (Fig. 4b). The improvement in the charge storage capacity of the NiCo_2_O_4_ electrode at higher temperature may be due to activation of the NiCo_2_O_4_ and sites and improved mobility of the electrolyte ions.

**Figure 5:**
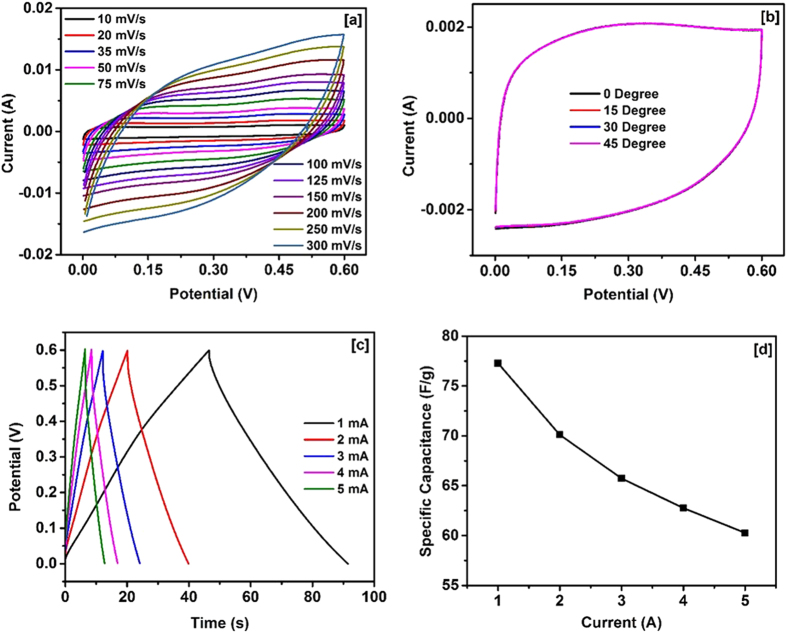
(**a**) CV curves at various scan rates, (**b**) CV curves at various bending angles, (**c**) Charge-discharge characteristics, and (**d**) Specific capacitance vs current plot for the device.

To understand the potential applicability of NiCo_2_O_4_ for flexible supercapacitor applications, we have fabricated a supercapacitor device by sandwiching an ion transporting layer between two NiCo_2_O_4_ electrodes. KOH was used as an electrolyte for this device. The electrochemical performances of the fabricated device were tested by measuring cyclic voltammetry curves and galvanostatic charge-discharge curves under various conditions. The CV curves of the device measured at various scan rates are shown in Fig. 5a. The rectangular shape and symmetry of the CV curves indicate near ideal capacitive nature of the fabricated device. As seen in the Fig. 5a, the CV curves of device are rectangular in shape without any significant redox waves, unlike that observed for the electrode, as the devices were constructed using two symmetrical electrode systems. This has also been noted for polyaniline-V_2_O_5_ composites^50^. The shape and symmetry of CV curves from the device is retained even at the high scan rates indicating high charge transfer stability of the device. It was also observed that the specific capacitance of the device decreases with increasing scan rate which could be due to insufficient time for electrochemical reactions at the electrode^51^. The flexibility of the device was tested by measuring CV curves at various bending angles (Fig. 5b). These CV curves were found to be very identical at several bending angles suggesting potential application of the device for flexible electronics. The charge storage capacity of the device was measured at different applied currents which are shown in Fig. 5c. Figure 5d shows the variation of specific capacitance with applied current. The decrease in the specific capacitance with increasing current could be due to increase of potential drop and insufficient faradic redox reaction at higher discharge currents. The Ragone plot of the device displaying variation in the energy density versus power density is shown in Figure 5S. As observed NiCo_2_O_4_ grown directly on conducting electrode display high energy and power densities.

**Figure 6:**
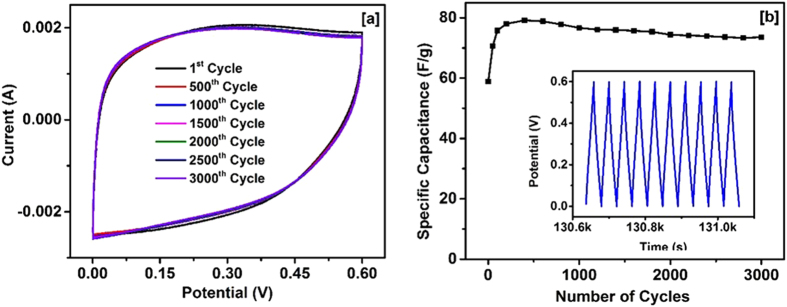
(**a**) CV curves of the NiCo_2_O_4_ supercapacitor device at different cycles, and (**b**) Variation of specific capacitance vs number of cycles for NiCo_2_O_4_ supercapacitor device. Inset figure shows last few cycles of charge-discharge characteristics of the device.

The long term cyclic stability of the device was electrochemically investigated. The cyclic stability of the device was measured for 3000 cycles. As seen in Fig. 6a, the device exhibited high cyclic stability with no sign of degradation in the charge storage capacity. Figure 6b shows the variation of specific capacitance versus number of cycles for the device. The specific capacitance of the device initially increases and then becomes almost constant (slight decrease with number of cycles). The inset of Fig. 6b shows the last few charge-discharge cycles of the device.

**Figure 7:**
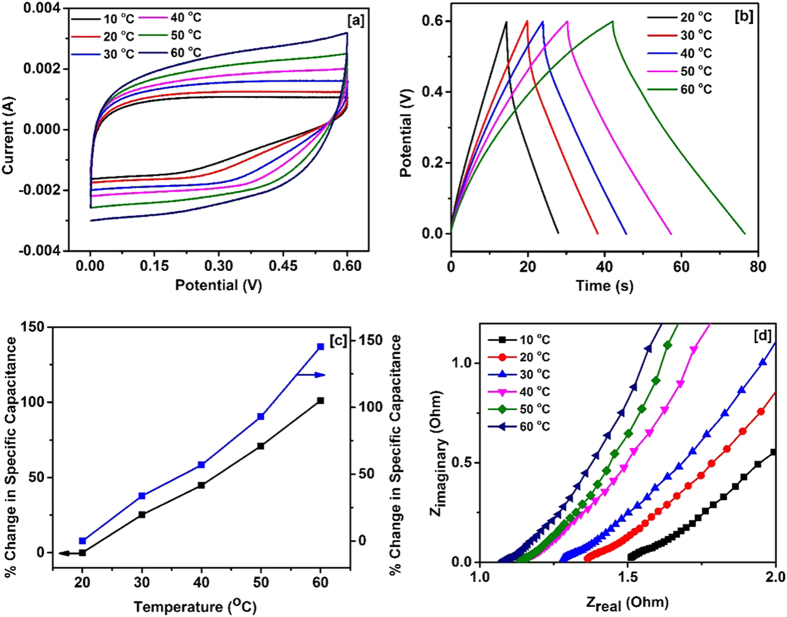
(**a**) CV curves at various temperatures, (**b**) Charge-discharge characteristics at various temperatures, (**c**) Change in specific capacitance with temperature derived from CV (black line) and charge-discharge characteristics (blue line), and (**d**) Variation of Z_real_ and I_imaginary_ at various temperatures for NiCo_2_O_4_ based supercapacitor device.

The temperature dependent electrochemical performance of the device was investigated using cyclic voltammograms, galvanostatic charge-discharge and electrochemical impedance measurements. The CV curves of the device are shown in Fig. 7a. The area under the CV curves was found to increase with increasing temperature, indicating enhanced charge storage capacity of the device at high temperature. In the charge-discharge study (Fig. 7b), the discharge time was observed to increase with increasing temperature, which further confirms higher capacity of the device at higher temperatures. The change in the specific capacitance versus temperature calculated using CV and charge-discharge measurements are shown in Fig. 7c. We observed about 150% improvement in the specific capacitance of the device by increasing temperature from 10 to 60 °C. The temperature dependent electrochemical behavior of the device was further analyzed using electrochemical impedance spectroscopy (EIS). Figure 7d shows the variation of real and imaginary impedance of the device at various temperatures. As observed, the equivalent series resistance (ESR) of the device (intercept at x-axis) decreases with increasing temperature which improves the charge storage capacity of the device. The decrease in the ESR value could be due to the enhanced mobility of the ions in the electrolyte which increases the conductivity of the electrolytes^52^.

## Conclusion

We have grown flower-like structures of NiCo_2_O_4_ that are composed of needle like crystallites using a facile hydrothermal method. The electrochemical properties of the NiCo_2_O_4_ electrode were studied using cyclic voltammetry, galvanostatic charge-discharge methods and electrochemical impedance spectroscopy. Distinctive pairs of redox waves were observed during the CV measurements attributing to Co (II)/Co (III) and Ni(II)/Ni(III) redox couples. The specific capacitance value as high as 845 F/g was obtained for the NiCo_2_O_4_ electrode and 77 F/g for the NiCo_2_O_4_ device in KOH electrolyte. The potential application of as developed NiCo_2_O_4_ electrode for flexible and operational in high temperature conditions was investigated. The CV and galvanostatic charge-discharge measurements on the device show high cyclic stability and flexibility. The charge storage capacity of the device was improved by ~150% by increasing temperature from 20 to 60 °C. The observed specific capacitance value of NiCo_2_O_4_ is considerably lower than those reported for the champion supercapacitor material RuO_2_. However, the cost of NiCo_2_O_4_ is insignificant in comparison with RuO_2_ for practical applications which make NiCo_2_O_4_ a promising material system with potential practical utility.

## Additional Information

**How to cite this article**: Gupta, R. K. *et al.* Flexible and High Performance Supercapacitors Based on NiCo_2_O_4_ for Wide Temperature Range Applications. *Sci. Rep.* **5**, 15265; doi: 10.1038/srep15265 (2015).

## Supplementary Material

Supplementary Information

## Figures and Tables

**Table 1:** Morphology and specific capacitance of NiCo_2_O_4_ grown using hydrothermal method.

**Morphology**	Specific capacitance(F/g)	**Reference**
Nanoflakes	844	53
Nanowall-network	1225	53
Chain-like nanowires	1284	54
Pine-like	2132	55
Nanowire arrays	2681	56
Flowerlike	658	36
Nanosheet	902	29
Nanoneedle	660	30
Nanosheet	891	42
Nanoneedle	845	This work

**Table 2:** Method used, substrate and specific capacitance of NiCo_2_O_4_.

**Method**	**Substrate**	Specific capacitance(F/g)	**Reference**
Electrochemical	Ni foam	835	31
Hydrothermal	Ni foam	2681	56
Hydrothermal	Ni foam	800	57
Hydrothermal	Ni foam	891	42
Electrodeposition	Ni foam	1450	58
Solution	Ni foam	1119	33
Electrochemical	Stainless steel	580	59
Chemical bath deposition	Indium tin oxide	490	60
Solution	Carbon fibers	1024	29
Electrodeposition	Carbon fibers	2658	61
Solvothermal	Carbon fibers	999	62
