# Supplementary material for: Flexible and High Performance Supercapacitors Based on NiCo2O4for Wide Temperature Range Applications
Source: Sci Rep. 2015 Oct 20;5:15265. doi: 10.1038/srep15265 (PMC4611180; doi:10.1038/srep15265)
Supplement: Supplementary Information [file srep15265-s1.pdf]

Flexible and High Performance Supercapacitors Based on $NiCo_2O_4$ for Wide Temperature Range Applications

Ram K. Gupta[a*], John Candler[a], Soubantika Palchoudhury[b], Karthik Ramasamy[c], Bipin Kumar Gupta[d]

[a] Department of Chemistry, Pittsburg State University, 1701 S. Broadway, Pittsburg, KS 66762, USA
[b] Center for Materials for Information Technology, The University of Alabama, Tuscaloosa, AL 35487, USA
[c] Center for Integrated Nanotechnologies, Los Alamos National Laboratory, Albuquerque, NM 87545, USA
[d] National Physical Laboratory (CSIR), Dr K.S. Krishnan Road, New Delhi 110012, India

Supplimentary Figures

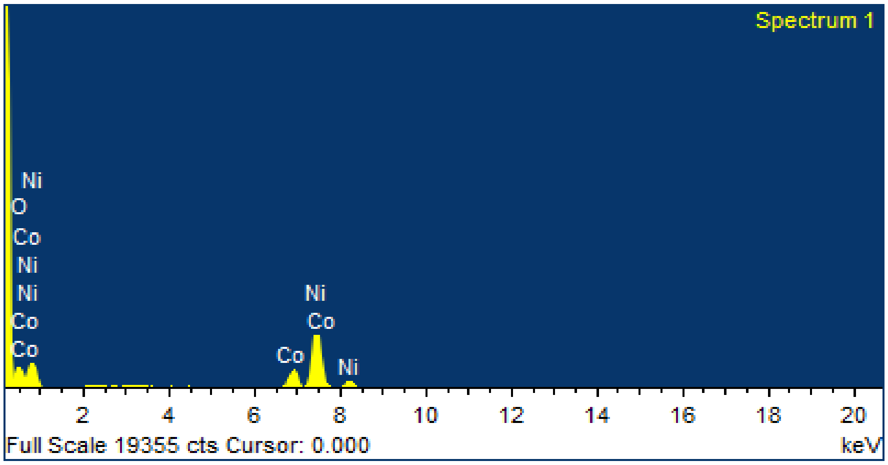

Fig. 1S: EDS images of the NiCo2O4 grown on nickel foam.

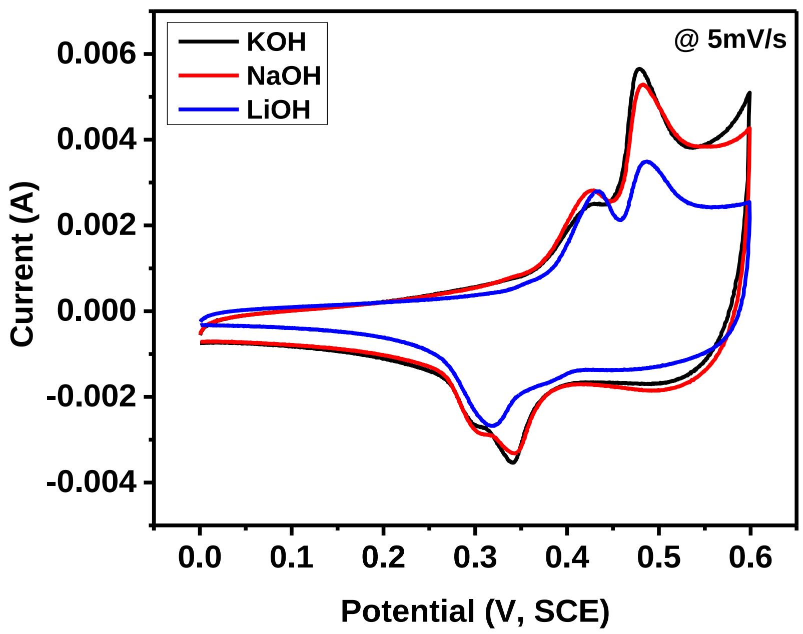

Fig. 2S: CV curves of $NiCo_2O_4$ in different electrolytes.

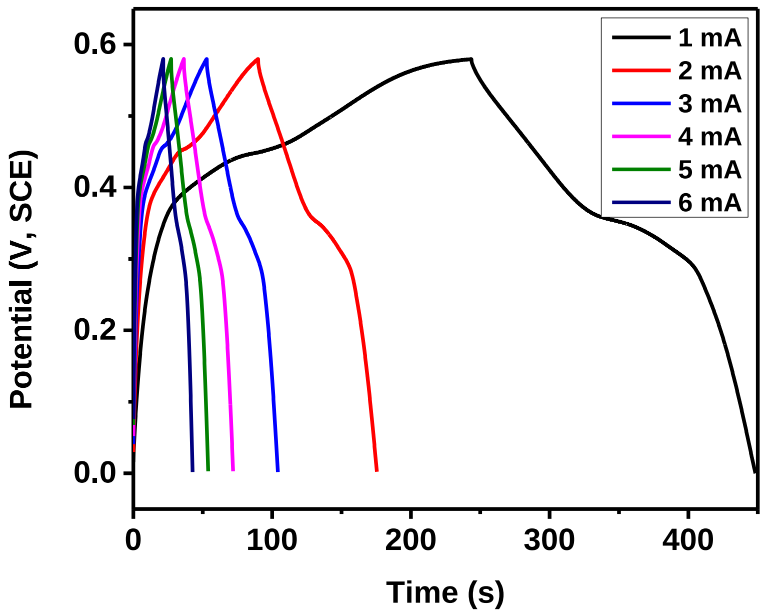

Fig. 3S: Charge-discharge characteristics of $NiCo_2O_4$ in 3M KOH electrolyte.

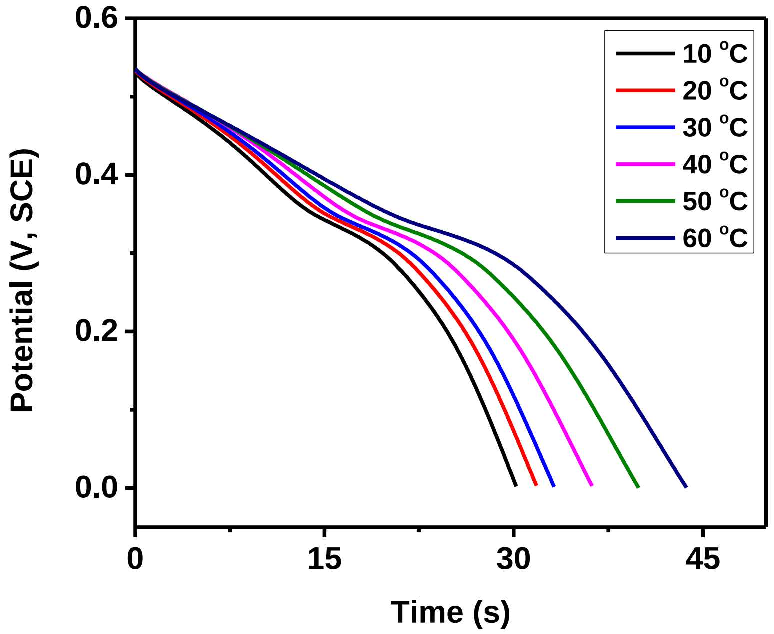

Fig. 4S: Discharge characteristics of $NiCo_2O_4$ electrode at various temperature.

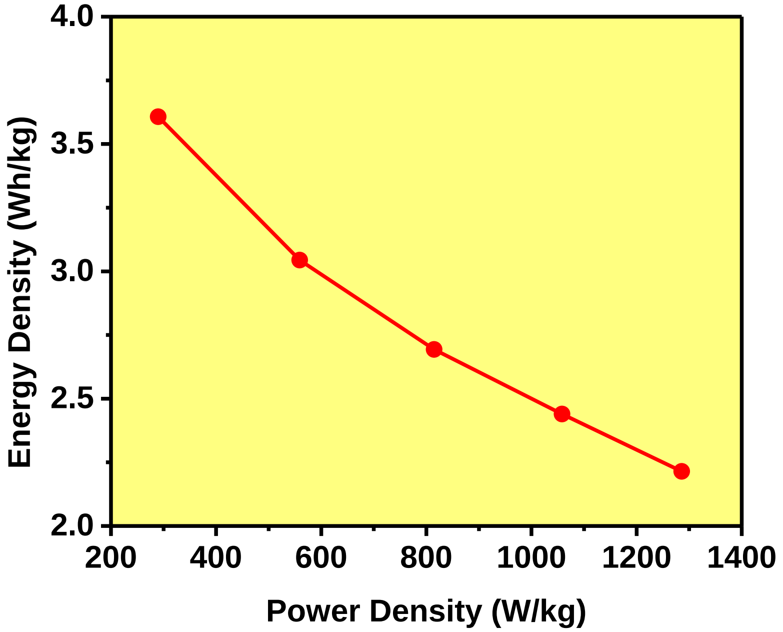

Fig. 5S: Ragone plot for $NiCo_2O_4$ based device.
